# Biomedical waste management practices and associated factors among health care workers in the era of the covid-19 pandemic at metropolitan city private hospitals, Amhara region, Ethiopia, 2020

**DOI:** 10.1371/journal.pone.0266037

**Published:** 2022-04-06

**Authors:** Getasew Mitiku, Amha Admasie, Amsalu Birara, Wubante Yalew

**Affiliations:** 1 Debretabor Health Science College, Debre Tabor, South Gondar, Ethiopia; 2 Department of Environmental Health, Bahir Dar University, School of Public Health, Bahir Dar, Ethiopia; 3 Public Health Researcher, Addis Ababa, Ethiopia; China University of Mining and Technology, CHINA

## Abstract

**Background:**

Biomedical waste management is an important precondition to safeguard the healthcare workers and community members, as well as the environment, from being contaminated with infectious substances. However, biomedical waste management practices during the pandemic era of COVID-19 were unknown.

**Objective:**

This study was aimed to assess biomedical waste management practices and associated factors among health care workers during the COVID-19 pandemic era at metropolitan city private hospitals, Amhara Region, Ethiopia.

**Methods:**

An institutional-based cross-sectional study was conducted at metropolitan city private hospitals in Amhara Region. Simple random sampling was used to select 431 study participants. Data were collected through a self-administered questionnaire and observational checklists. The data were cleaned, coded, and entered into the Epi-data version 4.6, and then exported to SPSS version 20. for analysis. Variables with a p-value less than 0.05 were considered as significant factors in multivariable logistic regression analysis and AOR with a 95% confidence level was used to measure the strength of association.

**Results:**

The proportion of health care workers who had good practices in biomedical waste management was 49.4%. Participants who had MSc education level, [AOR = 4.20, 95% CI (1.01, 17.40)], Bachelor degree [AOR = 3.52, 95% CI (2.13, 5.82)], got training on biomedical waste management [AOR = 4.33, 95% CI (2.71, 6.93)], access to color-coded three bins in their working department [AOR = 6.24.95% CI (3.84, 10.13)] and those who had good attitude (AOR = 2.64, 95% CI (1.65, 4.22), were significantly associated with biomedical waste management practices in private hospitals.

**Conclusion:**

The practice of biomedical waste management in the study area was low. Level of education, taking training on biomedical waste management, availability of color-coded three bins, and attitude of health care workers were significantly associated with biomedical waste management practices. Hence, in-service training is recommended to improve biomedical waste management practices.

## Introduction

Biomedical waste (BMW) is any waste that is generated during the diagnosis, treatment, or immunization of human beings or animals or from research activities, and contains potentially harmful microorganisms which will infect hospital communities and the general public [1,2].

BMW includes sharps, non-sharps, blood, body parts, chemicals, pharmaceuticals, medical devices, and radioactive materials [3]. Common sources of biomedical waste include hospitals, nursing homes, clinics, laboratories, offices of physicians, dental, and veterinarians, home health care, and funeral homes [4,5]. BMWs are considered because they represent the second hazardous waste globally after radiation waste [6].

Biomedical waste is a relevant problem for several countries and poses serious public health threats worldwide [7]. Nearly 3.2 million tons of biomedical waste is generated by hospitals alone annually and the Environmental Protection Agency (EPA.2019) estimates that 10% to 15% of all biomedical waste is potentially hazardous [8].

According to the World Health Organization (WHO), nearly 85% of waste generated by the hospitals is general waste and about 15% of waste is biomedical waste, composed of 10% of infectious wastes and 5% of non-infectious wastes like radioactive and chemical wastes [9]. In developing countries, especially in Africa, BMW has not received the attention it deserves [10].

Biomedical waste management (BMWM) is the process of segregation, collection, storage, treatment, transport and disposal, and other safety measures of waste in health institutions [11]. Proper BMWM includes vital steps, such as segregation, collection, storage, transportation, treatment, and final disposal, of waste generated in health care settings [12]. Improper BMWM, which includes hazardous wastes (10–25%) mixed with the non-hazardous waste (75–90%) can result in the whole bulk waste becoming potentially hazardous [13]. There are international agreements and Conventions which are particularly pertinent in BMWM, environment protection, and its sustainable development and thus they should be kept in mind by preparing waste management policies [14]. Adequate knowledge, attitude, and practice (KAP) of health care workers (HCWs) are key factors for having a successful BMWM system, as they are important preconditions to safeguard the community [15], and the environment from being contaminated with infectious substances [16].

In Ethiopia, public hospitals provide training associated with infection prevention and healthcare waste management to waste handlers, environmental professionals, and heads of departments, but there was no published evidence indicating that private hospitals provide any training associated with healthcare waste management and infection prevention for health care workers [17].

In the Ethiopian context, there was no separate regulation specific for the HCFs to enforce them for the proper management of hazardous waste. However there are three BWM guidelines prepared by the Federal Ministry of Health (FMoH), Food, Medicine and Healthcare Administration and Control Authority (FMHACA), and Federal Environmental Protection Authority (FEPA) independently which are not, updated and lacked proper compliance on their implementation[18–21].

COVID-19 has been reported to first begin in December 2019 [22] while the WHO announced a Global Pandemic in March 2020. COVID-19 has been rapidly spreading all over the world, forcing countries and governments to adopt strict and specific measures to contain the pandemic. According to the Federal Ministry of Health of Ethiopia, the first COVID-19 case was reported in March 2020, and measures for tackling the pandemic have been taken ever since. In this regard, proper disposal of the waste is strongly relevant, as it may lead to the spread of communicable diseases [23]. Abundant use of medical technologies in hospitals and safety measures to stop the dissemination of the COVID-19 have led to a tremendous increase in BMW generation [24]. The generation rate was reported about 9200 tons/day of PW, with a total generation of more than 3.3 million tons per year in India [25], and The total mean weight of waste generation rate in the hospital was 492.5 kg/day in Ethiopia [26]. Moreover, the waste generated in health care facilities during the treatment and laboratory tests is highly contagious and hazardous [23].

According to the WHO 2018 report, the biomedical waste generation rate in low-income countries was 0.2kg of hazardous waste per hospital bed per day [27]. However, the Biomedical waste generation rates vary across different hospitals in Ethiopia where the generation rate ranges from (0.164–1.94) kg/bed/ day, and (0.396–0.866) kg/bed day (0.92kg/bed/day and/or 0.75kg/) patient/day hazardous waste [28–31]. Health facilities in Ethiopia have chosen incineration to treat BMW [32,33], but 80% of hospital incinerators used low-temperature technology that generates air pollutants [34].

The BMW is often the source of over 30 dangerous blood-borne pathogens [35]. Worldwide, about 5.2 million people (including 4 million children) die each year due to exposure to BMW [36], The hazards of exposure to hospital waste can range from developing gastroenteritis, respiratory and skin infections, as well as more deadly diseases like Human Immunodeficiency Virus Acquired Immunodeficiency Syndrome (HIV/AIDS), and Hepatitis B (HBV); moreover, injections with contaminated syringes caused 21 million hepatitis B infections (32% of all new infections), 2 million hepatitis C (HCV) infections (40% of all new infections) and 260,000 HIV infections (5% of all new infections) [37,38].

In developing countries, the management of BMW is becoming a growing concern in urban areas [39]. However, Pathogens and toxic chemicals in BMW can pose serious health risks for waste collectors, patients, and health care workers. Among these risks, HIV/AIDS, HBV, and HCV can be mentioned. HIV, HCV, and HBV have the risk of transmission 0.3%, 1.8%, and 30%, respectively from one sharp injury [40].

Few studies conducted in Ethiopia indicated that lack of training, awareness, staff resistance, managerial poor commitment, lack of adequate resources, negligence, and unfavorable attitude of the healthcare staff were the main identified challenges of BMWM [21,28,41,42]. Therefore, assessing the practice of BWM and its associated factors among health care workers is a pivotal element to halting this burden. Accordingly, this study is planned to assess the practice of biomedical waste management and associated factors among health care workers in private hospitals of the metropolitan city of the Amhara region.

## Methods

### Study area

Amhara Region is found in Northwestern Ethiopia and has an estimated acreage of about 170000 square kilometers. The region borders Tigray within the North, Afar within the East, Oromiya within the South, Benishangul-Gumz within the Southwest, and also the country of Sudan to the West. The region has three metropolitan cities (Bahir Dar, Gondar, and Dessie). In line with the population size estimation of 2016, the total population was 1,937,081. (797,794 in Bahir Dar 740,859, in Gondar, and 398,428 in Dessie). In these metropolitan cities, there are eight private hospitals namely Gamby, Adinas, Afelas, Dreamcare, Ethiogeneral, Batty, Selam, and Ibex with six hundred ninety healthcare workers.

### Study design and period

An institutional-based cross-sectional study was carried out from November 25 to December 25/2020.

### Population

The source and study population of the study were all health care workers who were working in private hospitals in metropolitan cities of the Amhara region (Bahir Dar, Dessie, and Gondar). The study unit was, randomly selected health care workers.

### Inclusion and exclusion criteria

Health care workers in private hospitals who were employed 6 months or longer were included in the study, However, health care workers who were unable to communicate due to illness were not eligible for the study.

### Sample size determination and sampling procedure

The sample size was determined using Epi-info version 7 considering (78.9%) biomedical waste management practice in Debre Markos Town Healthcare Facilities, Amhara region [43]; at 4% of the marginal error, 95% of confidence level (CL), and a 10% response rate. Therefore, the sample size was 440. Amhara Region has three metropolitan cities. All private hospitals in the metropolitan cities in the region were identified by name and included in the study. The sample size was allocated proportionally to each private hospital. Then simple random sampling was employed to select healthcare workers from each private.

### Study variables

Biomedical waste management practice was our dependent variable. On the other side, socio-demographic characteristics of respondents, Healthcare facility-related factors, Knowledge of HCWs, and Attitude of HCWs were the independent variables of the study.

### Data collection method and instruments

The data were collected using a self-administered questionnaire and observational checklist. The questionnaire was comprised of socio-demographic characteristics, knowledge, attitude, and healthcare facility-related factors. The questionnaire and observational checklist were first developed in English and then translated into Amharic, by English and Amharic language professionals to check its consistency. Data were collected by 5 trained clinical nurses and supervised by 3 trained BSC Environmental Health Professionals.

### Quality control

The training was given to data collectors, and supervisors regarding the objective of the study, a basic skill of communication, how to conduct the self-administered questionnaire for one day. Before the actual data collection, pre-testing was conducted on 5% of the sample size at Debre Tabor Referral Hospital and the necessary correction was made based on the pre-testing findings. The completeness of the questionnaire was checked every day by the supervisors and principal investigator. These supervisors were available throughout the data collection period.

### Data processing and analysis

Data were entered into Epi-data software version 4.6 and then exported to the SPSS software version 20 for analysis. Descriptive statistics were carried out to illustrate the means, standard deviations, and frequencies of the demographic profile, knowledge, attitude, and BMWM practice. Binary logistic regression analysis was made to identify variables having an association with the dependent variable. Then all independent variables with a p-value < 0.25 in the bivariable analysis were entered into multivariable logistic regressions to control the effect of confounding. Model fitness was checked using the Hosmer Lemeshow test. Finally, variables with a p-value less than 0.05 were considered as significant factors, and AOR with a 95% confidence level was used to measure the strength of association.

### Ethical statement

Ethical clearance was obtained from the ethical review board of the college of medicine and health science, Bahir Dar University. Communication with different official administrators was done through a formal letter obtained from Bahir Dar University and the metropolitan cities health bureau. Before starting data collection, the participants had read the objective, benefits, and risks of the study to get informed verbal consent of participants. The right of the respondent to withdraw from the interview or not to participate was respected. To keep the confidentiality of any information provided by study participants, the data collection procedure was anonymous.

## Operational definition of terms

Biomedical waste, medical waste, healthcare waste, and hospital waste are terms that have been used interchangeably [41]. However, healthcare waste has been more frequently used by published articles so far [44].

### Biomedical waste management practice

The response to questions related to biomedical waste management practice was summed up and calculated the mean. The mean and above indicated good practice and the below mean indicated poor practice towards biomedical waste management practice [39].

### Knowledge

The response of knowledge questions was summed up and a total score was computed with value and taken mean score. The mean and above indicated good knowledge and the below mean indicated poor knowledge towards biomedical waste management practice [43].

**Attitude** is a judgment of individual behavior as good or poor and was measured based on the 5 points Likert scale by summing the Likert questions. The mean and above indicated a good attitude and the bellow mean indicated a poor attitude towards biomedical waste management practice [43].

### Health care workers

HCWs are people who are involved in the promotion, protection, and enhancement of population health. In this study, the term health care worker was standing for clinical staff and cleaners [43].

## Result

### Socio-demographic and healthcare-related characteristics

A total of 431 HCWs have participated in the study and the response rate was 98%. About, 245 (56.8%) were females. The mean age of the respondents was 29 years (with SD±4.68). Regarding educational status, 256 (59.4%) were first degree, and 12 (2.8%) were certificate and bellow. More than half, (52%) of the HCWs had more than 5 years of work experience. (Table 1).

**Table 1:** Socio-demographic variables for BMWM among HCWs at metropolitan cities private hospitals of Amhara region, Ethiopia, December 2020.

Variables	Response category	Frequency (n = 431)	Percent
Sex	Male	186	43.2
	Female	245	56.8
Age in years	<25	91	21.1
	26–30	203	47.1
	31–35	101	23.4
	>35	36	8.4
Religion	Orthodox	400	92.8
	Muslim	25	5.8
	Protestant	6	1.4
Marital status	Single	174	40.4
	Married	257	59.6
Ethnicity	Amhara	422	97.9
	Others	9	2.1
Level of education	MSc and above	15	3.5
	First Degree	256	59.4
	Diploma	148	34.3
	Certificate and bellow	12	2.8
Work experience	<1 year	50	11.6
	1–5 years	157	36.4
	>5 years	224	52.0

### Health care facility-related factors

Regarding training access, 201 (46.6%) of health care workers had taken BMWM training. About 388 (90%) workers were working 8 hours a day in different work environments such as 155 (36%) in OPD, 132 (30.6%) in Ward, and the rest in the laboratory, emergency, pharmacy, and others. In the working environment, only 223 (51.7%) of them had three bins for waste segregation. (Table 2)

**Table 2:** HCF related factors for BMWM among HCWs at metropolitan cities private hospitals of Amhara region, Ethiopia, 2020.

Variables	Response category	Frequency (n = 431)	Percent
Taken training on BMWM	Yes	199	46.2
	No	232	53.8
Working hours per day	8 hours	388	90.0
	>8 hours	43	10.0
Working departments	OPD	155	36.0
	Ward	132	30.6
	Laboratory	44	10.2
	Emergency	45	10.4
	Pharmacy	32	7.4
	Others**	23	5.3
Glove availability in working department	Yes	172	39.9
	No	151	35.0
	Not applicable	108	25.1
Guideline availability for BMWM or IPC	Yes	241	55.9
	No	190	44.1
Infectious waste stored more than two days	Yes	166	38.5
	No	265	61.5
Availability of 3 bins	Yes	223	51.7
	No	208	48.3
Leveled bins availability	Yes	371	86.1
	No	60	13.9

**x-ray and OR.

**Fig 1:**
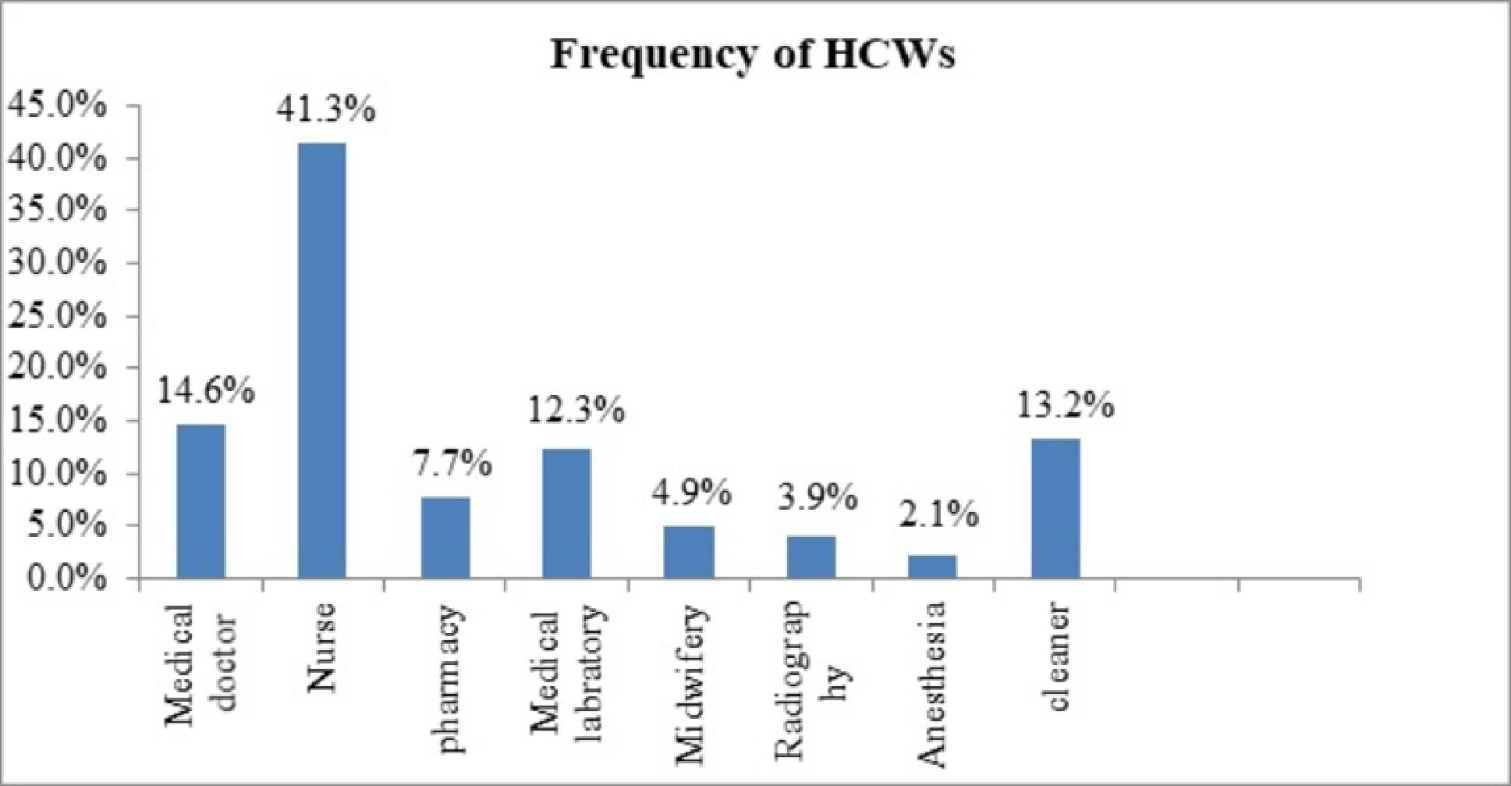
Profession or job distribution of health care workers in metropolitan cities private hospitals of Amhara region 2020.

As stated in Fig 1 below, among the studied participants, 178 (41.3%), 63 (14.6%)), and 58 (13.2%) were nurses, doctors, and cleaners respectively (Fig 1).

### Knowledge of health care workers

From the total health care workers, 290 (67.3%) HCWs knew the benefit of BMWM. About 269(62.4%), and 283(65.7%) were aware that infectious and general wastes, should be placed in yellow, and black, respectively. Besides, 233(54.1%) were aware of a safety box should be filled a maximum of 3/4^th^. 168 (39%) health care workers knew the maximum storage time (48 hours) limit of infectious wastes before treatment or disposal. Based on the summary of knowledge questions the mean score of HCW’s knowledge in biomedical waste management was 7.96 with SD±1.50 on a range of 1 to 13 questions. More than half, (62.4%) of Health care workers had good knowledge about biomedical waste management (Table 3).

**Table 3:** Frequency of health care workers among each knowledge term question at metropolitan cities private hospitals of Amhara region, Ethiopia, 2020.

Variables	Response category	Frequency (n = 431)	Percent
Know health hazards mainly associated with BMWs?	Yes	317	73.5
	No	114	26.5
Does wearing PPE reduce the risk of infection?	Yes	309	71.7
	No	122	28.3
Are all BMWs biologically hazardous (infectious)?	Yes	240	55.7
	No	191	44.3
Are items contaminated with body fluids considered as BMW?	Yes	315	73.1
	No	116	26.9
Do you know as BMWs are segregated into different categories?	Yes	304	70.5
	No	127	29.5
Do you know about the color-coding of BMW bins?	Yes	304	70.5
	No	127	29.5
What type of BMW should be stored in a yellow bin?	General waste	139	32.3
	Infectious waste	269	62.7
	I don’t know	23	5.3
What type of BMW should be stored in a black bin?	General waste	283	65.7
	Infectious waste	93	21.6
	I don’t know	55	12.8
What type of BMW should be stored in a safety box?	Sharp wastes	336	78.0
	Plastic wastes	67	15.5
	Paper wastes	28	6.5
Does disinfection of infectious BMWs decrease infection?	Yes	351	81.4
	No	80	18.6
Is there a need to close BMW containers while transport?	Yes	326	75.6
	No	105	26.4
Is there a need to secure stored BMWs waiting for treatment or disposal?	Yes	341	79.1
	No	90	20.9
Do you know about BMW disposal methods?	Yes	308	71.5
	No	123	28.5
Summary of BMWM Knowledge	Good knowledge	269	62.4
	Poor knowledge	162	37.6

### The attitude of health care workers

Among all Health care workers, 174 (40.4%) strongly agreed with the statement proper biomedical waste disposal is important and 167 (34.7%) health care workers strongly agreed with the statement BMWs should be segregated into different categories. Based on the summary of Attitude questions, the mean score of HCWs’ Attitude in biomedical waste management was 53.68 with SD±8.753 on a range of 1to 14 questions. More than half (53.4%) of Health care workers had a good attitude about biomedical waste management (Table 4).

**Table 4:** Frequency of health care workers among each attitude item question at metropolitan cities private hospitals of Amhara region, Ethiopia, 2020.

Variables	Disagree	Neutral	Agree
Improperly managed BMWs may cause infection	86(20.0)	42(9.7)	303(70.3)
Safe BMWM is an issue involving the responsibilities of every health care staff	40(9.3)	44(10.2)	347(80.5)
HIV may be transmitted through BMWs	68(15.8)	48(11.1)	315(73.1)
Hepatitis B virus may be transmitted through BMWs	64(14.8)	36(8.4)	331(76.8)
Hepatitis C virus may be transmitted through BMWs	72(16.7)	84(19.5)	275(63.8)
BMWs do not transmit any infectious diseases	275(63.8)	65(15.1)	91(21.1)
BMWs should be segregated into different categories at the point of generation	72(16.7)	52(12.1)	307(71.2)
BMW’s segregation facilitates safe handling of the waste	60(13.9)	36(8.4)	335(77.7)
Labeling BMWs containers does not add value to BMWM	251(58.2)	44(10.2)	136(31.6)
Proper biomedical waste disposal is important to prevent infection transmission	64(14.8)	12(2.8)	355(82.4)
BMW’s disinfection can reduce the chance of contracting an infection	62(14.4)	40(9.3)	329(76.3)
Wearing PPE helps to reduce the risk of infection	52(12.1)	48(11.1)	331(76.8)
BMW adds the extra burden of work	235(54.5)	72(16.7)	124(28.8)
Biohazards wastes should be disinfected before disposal	100(23.2)	36(8.4)	295(68.4)
Summary of BMWM Attitude score	Good attitude	230	53.4
	Poor attitude	201	46.6

### The practice of Health care workers

This study revealed that 98 (22.7%) health care workers encountered sharp injury at their health care service delivery. Regarding PPE, 337 (78.2%) and 332 (77.0%) of HCWs always used gloves and gowns while handling or working with BMWs respectively. Based on the summary of practice questions, the mean score of HCWs practice in this study was 6.77with SD ±1.42 on a range of 1 to 12 questions. Less than half (49.4%) of health care workers had a good practice of biomedical waste management with (95% CI: 44.6%, 54.2%) (Table 5).

**Table 5:** Practice of health care workers among each practice item question at metropolitan cities private hospitals of Amhara region, Ethiopia, December 2020.

Variables	Response category	Frequency	Percent
Sharp injuries	Yes	98	22.7
	No	333	77.3
Glove utilization	Yes	337	78.2
	No	94	21.8
Frequency of glove use	Always	272	63.1
	Sometimes	65	14.8
Do you wear a gown while you are working with/handling BMW?	Yes	332	77.0
	No	99	23.0
How often do you wear it?	Always	281	65.2
	Sometimes	51	11.8
Do you segregate BMW according to their type?	Yes	283	65.7
	No	148	34.3
Do you follow color coding for the segregation of BMWs?	Yes	283	67.7
	No	148	32.3
Where do you put non-infectious wastes like paper, plastic, and other supplies?	Black waste bin	252	58.5
	Yellow waste bin	140	32.5
	Safety box	39	9.0
Where do you put infectious wastes like cotton, gauze, and other items contaminated with blood and body fluids?	Black waste bin	110	25.5
	Yellow waste bin	257	59.6
	Safety box	64	14.8
Where do you put sharp wastes, which may cause punctures or cuts?	Black waste bin	44	10.2
	Yellow waste bin	68	15.8
	Safety box	319	74.0
Do you disinfect/decontaminate reusable devices after each use?	Yes	359	83.3
	No	72	16.7
Do you wash your hands with soap and water during your daily activity?	Yes	382	88.6
	No	49	11.4
Summary of BMWM practice	Good practice	213	49.4
	Poor practice	218	50.6

**Fig 2:**
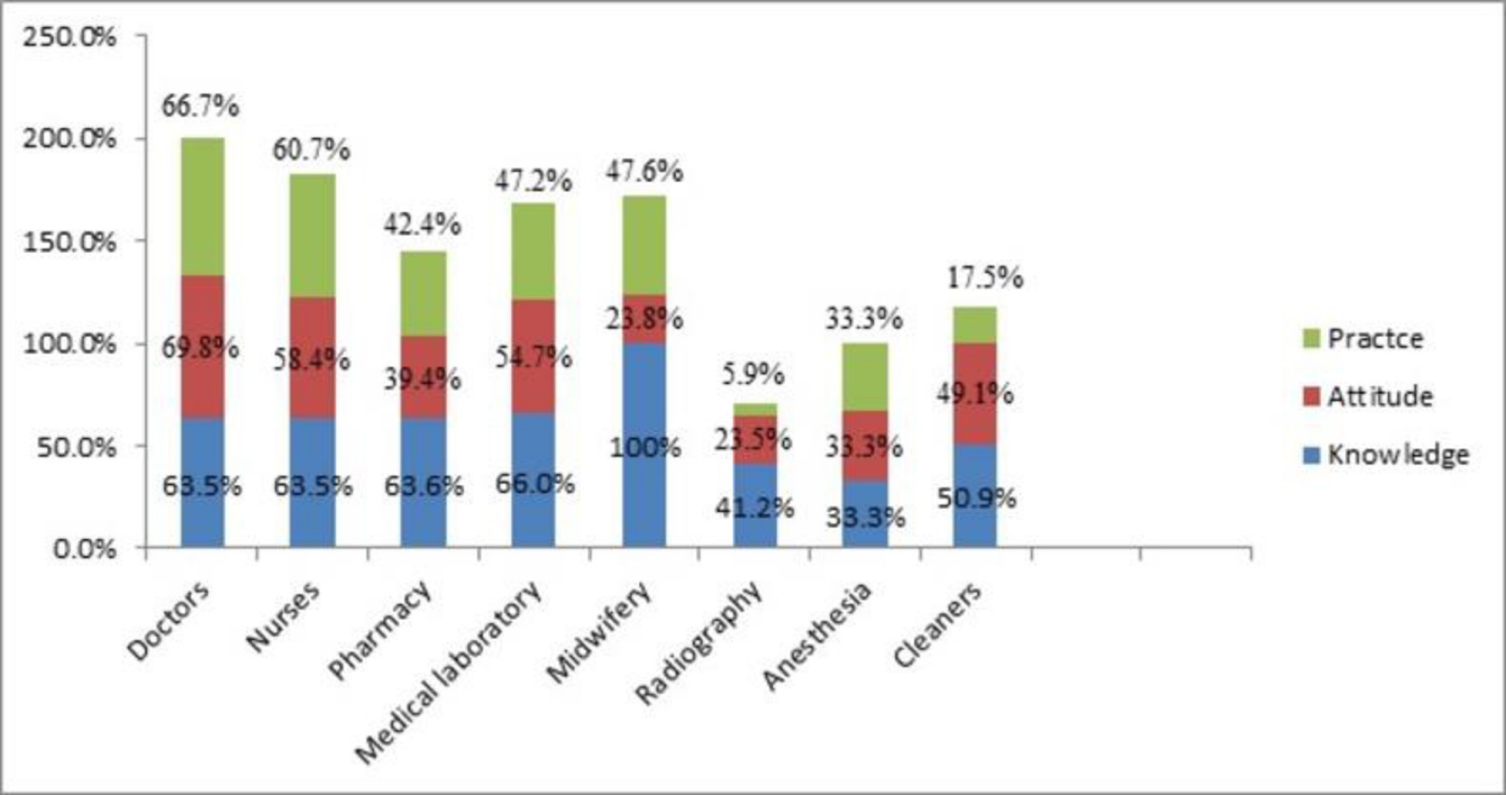
Frequency distribution of knowledge, attitude and practice of BMWM among HCWs in metropolitan cities private hospitals of Amhara region 2020.

Among the studied participants, HCWs who had high scores of biomedical waste management practice 66% and 60.7% were medical doctors and nurses respectively. whereas HCWs who had list scores of BMWM practice 17.5% and 5.9% were cleaners and radiographers respectively (Fig 2).

### Observation result

In the selected private hospital of each metropolitan city, observation was done at seven working departments such as OPDs, wards, laboratory, emergency, maternity, minor OR, pharmacy, and X-ray rooms of health care workers. Regarding the working department, more than half (62.5%) of departments had visual aid of biomedical waste containers. Gloves were available for each patient care cleaning device in all departments, except outpatient pharmacy departments. Three color-coding bins and leveled bins were available in Laboratory, Emergency, Maternity, and Minor OR departments, but not in other departments. The autoclave was available in some departments (maternity, Laboratory, and minor OR) but not in other departments rather it was available as a health care facility level in one fixed area. Personal protective equipment like heavy-duty gloves, aprons, and boots was available in maternity, emergency, laboratory, and minor OR rooms but not in others.

Regarding health care facilities, 37.5% of them had onsite storage rooms of biomedical wastes. The infection prevention and control committee was available only in two of them. All private hospitals had an incinerator, but it was not fenced (except one general hospital). Infection prevention and control guidelines were available in some hospitals’ infection prevention offices rather than in each working department. A placenta pit was available in all private hospitals.

### Factors associated with biomedical waste management practice

In the bi-variable binary logistic regression analysis; age, attitude, knowledge of HCWs, level of education, training, availability of three bins, information about biomedical waste, information about biomedical waste management, and work experience were factors associated with biomedical waste management practice.

To start with the findings of socio-demographic factor, the odds of good biomedical waste management practice was found to increase by more than 4 times among health care workers who hold MSc and above the level of education when compared with a diploma and below [AOR = 4.20, 95% CI: (1.01, 17.40)].

Health care workers who took training on biomedical waste management had an association with biomedical waste management practice. Health care workers who took training [AOR = 4.33, 95% CI: (2.71, 6.93)] were 4.3 times more likely to practice good biomedical waste management than their counterparts.

The availability of three bins (black bin, yellow bin, and safety box) in the working department was associated with good biomedical waste management practice. Availability of three bins in the working department [AOR = 6.24. 95% CI (3.84, 10.13)] was 6.2 times more likely to practice good biomedical waste management than not the availability of three bins.

Health care workers who had a good attitude [(AOR = 2.64, 95% CI: (1.65, 4.22] were 2.6 times more likely to practice good biomedical waste management than those who had a poor attitude (Table 6).

**Table 6:** Multivariable binary logistic regression analysis for factors associated with biomedical waste management practices among health care workers in metropolitan cities private hospitals of Amhara region, Ethiopia 2020.

Variables	BMWM practices		COR (95% CI)	AOR (95% CI)
	Good	Poor		
Age <25 years 26–30 years 31–35 years >35 years	35 107 49 22	56 96 52 14	1 1.78(1.08,2.95) 1.51(0.85,2.68) 2.51(1.14,5.55)	1 1.42(0.70.2.91) 0.77(0.34,1.74) 1.40(0.46,4.26)
Level of education MSc and above First degree Diploma and below	9 147 57	4 108 106	4.18(1.23,14.19) 2.53(1.68,3.80) 1	4.20(1.01,17.40) *** 3.52(2.13,5.82) *** 1
Taking training Yes No	139 74	60 158	4.95(3.28,7.45) 1	4.33(2.71,6.93) *** 1
Availability of three bins Yes No	154 59	69 149	5.64(3.73,8.53) *** 1	6.24(3.84,10.13) *** 1
Got information on BMWs Yes No	174 39	163 55	1.51(0.95,2.39) 1	0.75(0.37,1.54) 1
Got information on BMWM Yes No	176 37	156 62	1.89(1.19,2.99) * 1	1.61(0.91,2.86) 1
Attitude of HCWs Good Poor	140 73	90 128	2.73(1.85,4.03) *** 1	2.64(1.65,4.22) *** 1
Knowledge of HCWs Good Poor	139 74	130 88	1.27(0.86,1.88) 1	1.46(0.89,2.38) 1
Work experience <1 year 1–5 years >5 years	20 70 123	30 87 101	1 1.21(0.63,2.31) 1.83(0.98,3.41)	1 0.85(0.39,1.88) 1.46(0.68,3.14)

* = p-value<0.05

***p-value<0.001, Hosmer and Lemshow goodness of fit test = 0.839.

## Discussion

In this study, 213 (49.4%) health care workers had a good practice of BMWM with (95% CI: 44.6%, 54.2%). This finding is in line with the finding of two previous studies done in South Africa and Biyem- Assi District Hospital in Yaoundé, which reported 53.9% and 50% respectively [45,46]. However, the finding of this study is found to be higher than the findings of three studies done in Rwanda, Jigjiga, and Gondar town, which reported 33.5%, 42.3%, and 31.5 of good practices respectively. [39,47,48]. This disagreement might be partly explained by a difference in health facility setup., since the above-mentioned studies (Jigjiga and Gondar) had a mixing of hospitals and health centers and the other study (in Rwanda) had only one district hospital. But the current study included only general hospitals. So, hospitals might have good practice of BMWM due to the presence of health care workers who had a high level of education than the health centers. But, the finding of this study is found to be lower than the finding of other previous studies done at Debre Markos Town in Ethiopia, in a tertiary hospital in Puducherry (Southern India) and Mahatma Gandhi Government Hospital of India, which reported 78.9%, 69.3%, and 54.7% were found respectively [43,49,50]. The low level of practice shown in this study might be due to the more availability of 3 bins in 81.4% of health care workers in their working department at Debre Markos Town than the current studied health care workers (51.7%) and cultural differences of Indian health care setup and this local area.

In the present study, there was a significant association between the level of education and biomedical waste management practices. Health care workers who held MSc and above education level were 4.20 times more likely to practice good biomedical waste management than those who were diploma and below and health care workers who were degree level of education also were 3.52 times more likely to practice good biomedical waste management than those who were diploma and below. This finding was similar to the finding of a study done in the Capital city of Uganda [51]. This indicates that educational status development helps to improve the practice of health care workers on biomedical waste management [52].

The other finding worth highlighting is related to training, a significant association between taking training and biomedical waste management practice was found. Health care workers who took training on BMWM had 4.33 times more likely to practice good biomedical waste management than those who didn’t take the training. This finding was in agreement with the previous studies conducted in Gondar town, Ethiopia, and the capital city of Uganda [48,51]. It is due to getting waste management training of all those who are responsible for handling wastes is important to improve BMWM [53,54].

Availability of color-coded three bins was significantly associated with biomedical waste management practice. Health care workers who had three bins in their working department were 6.24 times more likely to practice biomedical waste management than those who had no three bins. The finding was supported by the previous study done in Debre Markos town, Ethiopia [43]. This is due to the availability of three bins that make waste segregation being simple and safe to separate hazardous wastes from non-hazardous general wastes [55].

The attitude of health care workers was significantly associated with biomedical waste management practice. Health care workers who had a good attitude toward BMWM had 2.64 times more likely to practice good biomedical waste management than those who had a poor attitude toward BMWM. This finding was supported by the studies done in Biyem- Assi District Hospital in Yaoundé (Cameroon) and Agartala, Tripura (North-eastern India) [45,56]. The possible explanation might be due to a good attitude of health care workers helps to practice good biomedical waste management; because the level of attitude was one of the factors, which affect practice as seen in other studies. The study was conducted in all metropolitan cities’ private hospitals of the Amhara region, which covered all private hospitals in three cities. But there may be socially desirable bias for the practice of BMWM during data collection time. In this study, the quantification of the generation rate of biomedical wastes should have been measured.

## Conclusion

Biomedical waste management practice was low among health care workers which is a risk of COVID 19 pandemic transmission. The level of education, taking training on BMWM, availability of three bins, and attitude of health care workers was found to have a significant association with biomedical waste management practice. Therefore, it was determined that it is better to provide in-service training programs on biomedical waste management and upgrade their educational level for health care professionals by regional health bureau and city administration health departments, as well as it is recommended to implement a three-bin system in the hospitals. Finally, all private hospitals should acknowledge the health care workers who practiced good biomedical waste management.

Although the study was conducted in private hospitals, the health tier system in Ethiopia both for private and public Hospitals is similar except for the ownership. Therefore, the finding can apply to other similar public hospitals within and across regions as well as in the least and middle-income countries.

## Supporting information

S1 File Data collection tool English version. (PDF)

S2 File Data collection tool Amharic version. (PDF)

S3 File Data. (XLSX)

## Abbreviations

AIDS: Acquired Immunodeficiency Syndrome
BMW: Biomedical waste
BMWM: Biomedical waste management
HBV: Hepatitis B Virus
HCB: Hepatitis C Virus
HCWs: Health Care Workers
HIV: Human Immunodeficiency Virus
IPC: Infection Prevention and Control
NGOs: Non-Governmental Organization
PPE: Personal Protective Equipment
SOP: Standard Operating Procedure
WHO: World Health Organization
