# Supplementary material for: Biomedical waste management practices and associated factors among health care workers in the era of the covid-19 pandemic at metropolitan city private hospitals, Amhara region, Ethiopia, 2020
Source: PLoS One. 2022 Apr 6;17(4):e0266037. doi: 10.1371/journal.pone.0266037 (PMC8985930; doi:10.1371/journal.pone.0266037)
Supplement: S1 File — (PDF) [file pone.0266037.s001.pdf]

Annex IV: - Amharic version questionnaire

ክፍል 1፡-ማህበራዊና ስነ ሕዝባዊ መጠይቆች

ተ.ቁ	መጠይቅ	መልስ
101	ጾታ	1. ወንድ<br>2. ሴት
102	ዕድሜ	………… ዓመት
103	ሃይማኖት	1. ኦርቶዶክስ<br>2. ሙስሊም<br>3. ፕሮቴስታንት<br>4. ካቶሊክ<br>5. ሌሎች (ይገለጽ)……….
104	የጋብቻ ሁኔታ	1. ያላገባ/ያላገባች<br>2. ያገባ/ ያገባች<br>3. ባሏ የሞተ / ሚስቱ የሞተች<br>4. ትዳር የፈታች /የፈታ<br>5. 5. በጓደኝነት(ከትዳር ውጭ)የሆነ/የሆነች
105	ብሄር	1. አማራ<br>2. ትግራይ<br>3. ኦሮሞ<br>4. አፋር<br>5. ሌሎች (ይገለጽ)
106	የትምህርት ደረጃ	1. ማስተር እና ከዚያ በላይ<br>2. ዲግሪ<br>3. ዲፕሎማ<br>4. ሰርቲፊኬት እና ከዚያ በታች
107	የሙያ ዘርፍ	1. ሀኪም<br>2. አዋላጅ ነርስ<br>3. ነርስ<br>4. ራዲዮግራፊ<br>5. ፋርማሲ<br>6. አንስቴዥያ<br>7. ላብራቶሪ<br>8. ጽዳተኛ

		9. የአካባቢ ጤና አጠባበቅ ባለሙያ<br>10.10.ሌሎች (ይገለጽ)_____
108	የስራ ልምድ	___________ዓመት/ወር
109	በወር የሚያገኙት የደመወዝ መጠን ምን ያህል ነዉ?	_______ ብር

ክፍል 2፡ ከጤና ተቋማት ጋር ተያያዥነት ያላቸዉ መጠይቆች

ተ.ቁ	መጠይቅ	መልስ	
201	በተቋሙ የባዮሜዲካል ቆሻሻ አያያዝ ወይም ተዛማጅ ጉዳዮች ላይ ሥልጠና ወስደው ያውቃሉ?	1.አዎ	2.የለም
202	በሙያዎ በቀን ስንት ሰዓት ይሰራሉ?	_____	
203	በሙያዎ በሳምንት ስንት ቀን ይሰራሉ?	_____	
204	አሁን የሚሠሩት በየትኛው የህክምና ክፍል ነዉ?	1. ተመላላሽ<br>2. ፋርማሲ<br>3. ተኝቶ ህክምና<br>4. ቀዶ ጥገና<br>5. ላብራቶሪ<br>6. ድንገተኛ<br>7. ቢሮ<br>8. ሌሎች (ይገለጽ)_____	
205	በሚሰሩበት ክፍል ለእያንዳንዱ በሽተኛ ህክምና አገልግሎት የሚዉሉ ጓንቶች በበቂ ይገኛሉ?	1. አዎ<br>2. የለም<br>3. አስፈላጊ አይደለም	
206	በሚሰሩበት ክፍል የባዮሜሚካል ቆሻሻ አያያዝ ወይም የብክለት መከላከያ እና መቆጣጠሪያ የስራ ሰነድ አለ?	1. አዎ<br>2. የለም	
207	በሚሰሩበት ክፍል የባዮሜዲካል ቆሻሻ አያያዝን ወይም ብክለትን ለመከላከል የሚያስችል መደበኛ የአሠራር ዘዴ መመሪያ አለ?	1. አዎ<br>2. የለም	
208	በሚሰሩበት ክፍል ከሁለት ቀናት በላይ የተከማቸ በሽታ አስተላላፊ ቆሻሻ አለ?	1. አዎ<br>2. የለም	
209	ተቋሙ በቦታው ላይ የባዮሜዲካል ቆሻሻ ማከሚያ ዘዴዎችን ይጠቀማል?	1. አዎ<br>2. የለም	

210	በጥያቄ 209 መልስዎ አዎ ከሆነ ተቋሙ ምን ዓይነት ባዮሜዲካል ቆሻሻ ማከሚያ ዘዴ አለዉ? (ብዙ መልሶች ይቻላል)	1. በኢንሲኔረተር ማቃጠል<br>2. ማምከን<br>3. በኬሚካል ማከም<br>4. ከጉድጓድ ማቃጠል<br>5. ሌላ (ይግለጹ) ______
211	ሁሉም 3ቱ ባዮሜዲካል ቆሻሻ መያዣዎች (ጥቁር ፣ ቢጫ እና የደህንነት ሳጥን) በሚሰሩበት ክፍል ውስጥ ይገኛሉ?	1. አዎ<br>2. የለም
212	ግልጽ ምልክት የተደረገባቸዉ የባዮሜዲካል ቆሻሻ መያዣ እቃዎች በሚሰሩበት ክፍል ውስጥ ይገኛሉ?	1. አዎ<br>2. የለም

ክፍል 3፡ የጤና እንክብካቤ ሰራተኞች ዕውቀትን ለመዳሰስ የተዘጋጁ ጥያቄዎች

ተ.ቁ	መጠይቆች	መልስ
301	ስለ ባዮሜዲካል ቆሻሻ ሰምተዉ ያውቃሉ?	1. አዎ<br>2. የለም
302	በጥያቄ 301 መልስዎ አዎ ከሆነ ከየት አገኙት?<br>(ከአንድ መልስ በላይ መምረጥ ይችላሉ)	1. ከሥራመመሪያ ሰነድ<br>2. ከስልጠና<br>3. ከጓደኞች<br>4. ከቴሌቪዥን<br>5. ከሬዲዮ<br>6. ከጋዜጣ<br>7. ሌሎች (ይግለጹ) _____
303	ስለ ባዮሜዲካል ቆሻሻ አያያዝ ሰምተዉ ያውቃሉ?	1. አዎ<br>2. የለም
304	በጥያቄ 303 መልስዎ አዎ ከሆነ ከየት አገኙት?<br>(ከአንድ መልስ በላይ መምረጥ ይችላሉ)	1. ከሥራ መመሪያ ሰነድ<br>2. ከስልጠና<br>3. ከጓደኞች<br>4. ከቴሌቪዥን<br>5. ከሬዲዮ<br>6. ከጋዜጣ<br>7. ሌሎች (ይግለጹ) _____
305	የባዮሜዲካል ቆሻሻዎች እንደገና ጥቅም ላይ እንደሚዉሉ ሰምተዉ ያውቃሉ?	1. አዎ<br>2. የለም

306	በጥያቄ 305 መልስዎ አዎ ከሆነ ከየት አገኙት?<br><br>(ከአንድ መልስ በላይ መምረጥ ይችላሉ)	1. ከሥራ መመሪያ ሰነድ<br>2. ከስልጠና<br>3. ከጓደኞች<br>4. ከቴሌቪዥን<br>5. ከሬዲዮ<br>6. ከጋዜጣ<br>7. ሌሎች (ይግለጹ) _____
307	ስለ ባዮሜዲካል ቆሻሻ አያያዝ ጥቅም ሰምተዉ ያውቃሉ?	1. አዎ<br>2. የለም
308	በጥያቄ 307 መልስዎ አዎ ከሆነ ከየት አገኙት?<br><br>(ከአንድ መልስ በላይ መምረጥ ይችላሉ)	1. ከሥራ መመሪያ ሰነድ<br>2. ከስልጠና<br>3. ከጓደኞች<br>4. ከቴሌቪዥን<br>5. ከሬዲዮ<br>6. ከጋዜጣ<br>7. ሌሎች (ይግለጹ) _____
309	ከባዮሜዲካል ቆሻሻ ጋር የተዛመደ የጤና አደጋ መኖሩን ያውቃሉ?	1. አዎ<br>2. የለም
310	በጥያቄ 309 መልስዎ አዎ ከሆነ ምን አይነት የጤና አደጋ ያውቃሉ? (ከአንድ መልስ በላይ መምረጥ ይችላሉ)	1. የኤች.አይ.ቪ. ቫይረስ ብክለት<br>2. የሄፓታይተስ ቢ ቫይረስ ብክለት<br>3. የሄፓታይተስ ሲ ቫይረስ ብክለት<br>4. ሌላ (ይግለጹ) ____
311	የግል ደህንነት መጠበቂያ መሳሪያዎችን መልበስ ለብክለት የመጋለጥ እድልን ይቀንሳል ብለው ያስባሉ?	1. አዎ<br>2. የለም
312	በጥያቄ 311 መልስዎ አዎ ከሆነ ለምን አይነት ብክለት የመጋለጥ እድልን ይቀንሳሉ? (ከአንድ መልስ በላይ መምረጥ ይችላሉ)	1. ኤች.አይ.ቪ. ቫይረስ<br>2. ሄፓታይተስ ቢ ቫይረስ<br>3. ሄፓታይተስ ሲ ቫይረስ<br>4. ሌላ (ይግለጹ) ____
313	ሁሉም ባዮሜዲካል ቆሻሻዎች ለህይወት አደጋ ናቸው ብለው ያስባሉ?	1. አዎ<br>2. የለም

314	በሰውነት ፈሳሽ የተበከሉ ነገሮች ከባዮሜዲካል ቆሻሻ ይመደባሉ ብለው ያስባሉ ?	1. አዎ<br>2. የለም
315	የባዮሜዲካል ቆሻሻዎች ወዲያዉኑ እንደ ተፈጠሩ ወደ ተለያዩ ምድቦች መከፋፈል አለባቸው ብለው ያስባሉ?	1. አዎ<br>2. የለም
316	ስለ ባዮሜዲካል ቆሻሻ ማስቀመጫ ዕቃ መለያ ቀለም ያውቃሉ?	1. አዎ<br>2. የለም
317	በጥያቄ 316 መልስዎ አዎ ከሆነ ምን ዓይነት የቆሻሻ ማስቀመጫ ዕቃ መለያ ቀለም ያውቃሉ? (ከአንድ መልስ በላይ መምረጥ ይችላሉ)	1. ቢጫ የቆሻሻ ማጠራቀሚያ<br>2. ጥቁር የቆሻሻ ማጠራቀሚያ<br>3. ቢጫ የደህንነት ሳጥን<br>4. ሌላ (ይግለጹ) ____
318	በቢጫ የባዮሜዲካል ቆሻሻ ማጠራቀሚያ ቅርጫት ውስጥ መጣል ያለበት ምን ዓይነት ባዮሜዲካል ቆሻሻ ነዉ?	1. ያልተበከለ ቆሻሻ<br>2. የተበከለ ቆሻሻ<br>3. አላውቅም
319	በጥቁር የባዮሜዲካል ቆሻሻ ማጠራቀሚያ ቅርጫት ውስጥ መጣል ያለበት ምን ዓይነት ባዮሜዲካል ቆሻሻ ነዉ?	1. ያልተበከለ ቆሻሻ<br>2. የተበከለ ቆሻሻ<br>3. አላውቅም
320	በደህንነት ሳጥን ውስጥ መጣል ያለበት ምን ዓይነት ባዮሜዲካል ቆሻሻ ነዉ?	1. የስለታማ ነገሮች ቆሻሻ<br>2. የፕላስቲክ ቆሻሻ<br>3. የወረቀት ቆሻሻ<br>4. ሌላ (ይግለጹ) ____
321	ለህክምና ያገለገሉ ስለታማ የሕክምና እቃዎችን የያዘ የደህንነት ሳጥን ምን ያህል ሙሉ መሆን አለበት ብለው ያስባሉ?	1. 1/2 ኛው<br>2. ¾ ኛው<br>3. ሙሉ ለሙሉ<br>4. አላውቅም
322	ስለ ባዮሜዲካል ቆሻሻ ማስቀመጫ ዕቃ መሰየምን ያውቃሉ?	1. አዎ<br>2. የለም
323	በጥያቄ 322 መልስዎ አዎ ከሆነ ምን ዓይነት ስያሜ ያውቃሉ?<br>(ከአንድ መልስ በላይ መምረጥ ይችላሉ)	1. የተበከሉ ቆሻሻዎች መያዣ<br>2. ያልተበከሉ ቆሻሻዎች መያዣ

		3. ስለታማ ቆሻሻዎች መያዣ<br>4. ሌሎች (ይግለጹ)___
324	በሽታ አስተላላፊ የባዮሜዲካል ቆሻሻዎችን ማከም ብክለትን ይቀንሳል ብለው ያስባሉ?	1. አዎ<br>2. የለም
325	የባዮሎጂካል ቆሻሻ ማጠራቀሚያዎችን በምናጓጉዝበት ጊዜ መዝጋት ያስፈልጋል ብለው ያስባሉ?	1. አዎ<br>2. የለም
326	የተከማቹ ባዮሜዲካል ቆሻሻዎችን ታክመዉ እስኪወገዱ ድረስ ጠብቆ ማቆየት ያስፈልጋል ብለው ያስባሉ?	1. አዎ<br>2. የለም
327	በሽታ አስተላላፊ የባዮሜዲካል ቆሻሻን ከማከምዎ ወይም ከማስወገድዎ በፊት ቢበዛ ምን ያህል ጊዜ ይቆያል?	1. 24 ሰዓት<br>2. 72 ሰዓት<br>3. 48 ሰዓት<br>4. አላውቅም
328	ስለ ባዮሜዲካል ቆሻሻ አወጋገድ ዘዴዎች ያውቃሉ?	1. አዎ<br>2. የለም
329	በጥያቄ 328 መልስዎ አዎ ከሆነ ምን ዓይነት ማስወገጃ ዘዴዎችን ያዉቃሉ? (ከአንድ መልስ በላይ መምረጥ ይችላሉ)	1. የአመድ ጉድጓድ<br>2. የመርፌ ማስወገጃ<br>3. በመቅበር<br>4. ሌላ(ይግለጹ) _______

ክፍል 4፡ ከጤና እንክብካቤ ሰራተኞች አመለካከት ጋር የተዛመዱ ጥያቄዎች ።

በሚከተለው የመጠን ልኬት 1-5 ላይ የተመሠረተ (1 = በፍፁም አልስማማም ፣ 2 = አልስማማም፣ 3 = እርግጠኝ አይደለሁም/አላውቅም፤ 4 = እስማማለሁ እና 5 = በጣም እስማማለሁ። እባክዎን እያንዳንዱን መግለጫ ያንብቡ እና መልስዎን ከሚያምኑት ሰንጠረዥ በስተቀኝ ህዳግ ላይ ይምረጡ ።

ተ.ቁ	በሚቀጥሉት መግለጫዎች ላይ አስተያየትዎ / እምነትዎ ምንድነው?	በፍፁም አልስማማም	አልስ ማማ ም	እርግጠ ኛ<br>አይደለ ሁም/አላ ውቅም	እስ ማማ ለሁ	በጣ ም እስ ማ ማለ ሁ

401	በአግባቡ ያልተከናወኑ (ያልተያዙ) ባዮሜዲካል ቆሻሻዎች ብክለትን ሊያስከትሉ ይችላሉ፡፡	1	2	3	4	5
402	ደህንነቱ የተጠበቀ የባዮሜካኒካል ቆሻሻ አያያዝ የእያንዳንዱን የጤና እንክብካቤ ሰራተኛ ሃላፊነት የሚመለከት ጉዳይ ነው፡፡	1	2	3	4	5
403	ኤች አይ ቪ ቫይረስ በባዮሜዲካል ቆሻሻዎች በኩል ሊተላለፍ ይችላል፡፡	1	2	3	4	5
404	የሄፓታይተስ ቢ ቫይረስ በባዮሜዲካል ቆሻሻዎች ሊተላለፍ ይችላል፡፡	1	2	3	4	5
405	የሄፓታይተስ ሲ ቫይረስ በባዮሜዲካል ቆሻሻዎች ሊተላለፍ ይችላል፡፡	1	2	3	4	5
406	የባዮሜዲካል ቆሻሻዎች ማንኛውንም ተላላፊ በሽታ አያስተላልፉም፡፡	1	2	3	4	5
407	የባዮሜዲካል ቆሻሻዎች ወዲያዉኑ እንደተፈጠሩ በየደረጃቸዉ ወደ ተለያዩ ምድቦች መከፋፈል አለባቸው፡፡	1	2	3	4	5
408	የባዮሜዲካል ቆሻሻን መለየት ቆሻሻውን በጥንቃቄ ለመያዝ ይረዳል፡፡	1	2	3	4	5
409	የባዮሜዲካል ቆሻሻ ማጠራቀሚያዎችን መሰየሙ በባዮሜዲካል ቆሻሻ አያያዝ ላይ ጥቅም የለዉም፡፡	1	2	3	4	5
410	የብክለት ስርጭትን ለመከላከል ትክክለኛ የባዮሜዲካል ቆሻሻ አወጋገድ አስፈላጊ ነው፡፡	1	2	3	4	5
411	የባዮሜዲካል ቆሻሻን ማከም የመበከል እድልን ሊቀንስ ይችላል፡፡	1	2	3	4	5
412	የግል ደህንነት መከላከያ መሳሪያዎችን መጠቀሙ ለብክለት የመጋለጥ እድልን ይቀንሳል፡፡	1	2	3	4	5
413	የባዮሜዲካል ቆሻሻ አያያዝ ተጨማሪ የሥራ ጫና ይጨምራል፡፡	1	2	3	4	5
414	የባዮሜዲካል ቆሻሻዎች አያያዝ የተቋሙ ኃላፊነት ብቻ ነው፡፡	1	2	3	4	5

415	ለህይወት አደገኛ የሆኑ ቆሻሻዎች ከመወገዳቸው በፊት መታከም አለባቸው።	1	2	3	4	5

ክፍል 5፡ከጤና እንክብካቤ ሰራተኞች ተግባር ጋር የተዛመዱ ጥያቄዎች።

ተ.ቁ	መጠይቅ	መልስ
501	ባለፉት 6 ወራት ማንኛውም የስለታማ ነገር ጉዳት አጋጥሞዎት ያውቃል?	1. አዎ<br>2. የለም
502	በጥያቄ 601 መልስዎ አዎ ከሆነ ምን ዓይነት የስለት ጉዳት ነዉ?<br>(ከአንድ መልስ በላይ መልስ መምረጥ ይቻላሉ)	1. የቀዶጥገና ማድረጊያ ስለት<br>2. ምላጭ<br>3. መርፌ<br>4. ሌሎች(ይግለጹ)------
503	ባዮሜዲካል ቆሻሻዎችን በሚይዙበት ጊዜ ጓንት ይጠቀማሉ?	1. አዎ<br>2. የለም
504	በጥያቄ 503 መልስዎ አዎ ከሆነ ምን ያህል ጊዜ? (ሁል ጊዜ፡ አስፈላጊ በሚሆኑበት በቀጣይነት መጠቀም፣ አንዳንድ ጊዜ፡- አስፈላጊ በሚሆኑበት ጊዜ አልፎ አልፎ መጠቀም በጭራሽ፡- አስፈላጊ በሚሆኑበት ጊዜ አለመጠቀም)	1. ሁል ጊዜ<br>2. አንዳንድ ጊዜ<br>3. በጭራሽ
505	ከባዮሜዲካል ቆሻሻዎች ጋር አብረው በሚሠሩበት / በሚይዙበት ጊዜ ጋዎን ይለብሳሉ?	1. አዎ<br>2. የለም
506	በጥያቄ 505 መልስዎ አዎ ከሆነ ምን ያህል ጊዜ?	1. ሁል ጊዜ<br>2. በጭራሽ<br>3. አንዳንድ ጊዜ
507	የባዮሜዲካል ቆሻሻ ማጠራቀሚያዎችን ይሰይማሉ?	1. አዎ<br>2. የለም
508	በጥያቄ 507 መልስዎ አዎ ከሆነ እንዴት ይሰይማሉ? (ከአንድ መልስ በላይ መልስ መምረጥ ይቻላሉ)	1. የተበከሉ ቆሻሻዎች መያዣ<br>2. ያልተበከሉ ቆሻሻዎች መያዣ<br>3. የስለታማ ቆሻሻዎች መያዣ<br>4. ሌሎች(ይግለጹ)____
509	የባዮሜዲካል ቆሻሻዎችን እንደተመረቱ በየደረጃቸው ዓይነት ይለያሉ?	1. አዎ

		2. የለም
510	ለባዮሜዲካል ቆሻሻዎች መያዣ የቀለም መለያ ኮድ ይከተላሉ?	1. አዎ<br>2. የለም
511	በጥያቄ 510 መልስዎ አዎ ከሆነ ምን ዓይነት የቀለም ኮድ መለያ ይከተላሉ? (ከአንድ መልስ በላይ መምረጥ ይችላሉ)	1. ጥቁር የቆሻሻ መያዣ<br>2. ቢጫ የቆሻሻ መያዣ<br>3. ቢጫ የደህንነት ሳጥን<br>4. ሌላ (ይግለጹ) _______
512	በሽታ አስተላላፊ ያልሆኑ ቆሻሻዎችን እንደ ወረቀት ፣ ፕላስቲክ እና ሌሎች አቅርቦቶችን የት ያስቀምጣሉ?	1. ጥቁር የቆሻሻ መያዣ<br>2. ቢጫ የቆሻሻ መያዣ<br>3. የደህንነት ሳጥን<br>4. ሌላ (ይግለጹ) _______
513	እንደ ጥጥ ፣ ፋሻ እና ሌሎች በደም እና በሰውነት ፈሳሾች የተበከሉ በሽታ አስተላላፊ ቆሻሻዎችን የት ያኖራሉ?	1. ጥቁር የቆሻሻ መያዣ<br>2. ቢጫ የቆሻሻ መያዣ<br>3. የደህንነት ሳጥን<br>4. ሌላ (ይግለጹ) _______
514	መወጋትን ወይም መቆረጥን ሊያስከትሉ የሚችሉ የህክምና ቁሳቁሶችን የት ያኖራሉ?	1. ጥቁር የቆሻሻ መያዣ<br>2. ቢጫ የቆሻሻ መያዣ<br>3. የደህንነት ሳጥን<br>4. ሌላ(ይግለጹ) _______
515	ጊዜ ያለፈበትን መድኃኒት የት ያስቀምጣሉ?	1. ጥቁር የቆሻሻ መያዣ<br>2. ቢጫ የቆሻሻ መያዣ<br>3. የደህንነት ሳጥን<br>4. የለም(አላገኘሁም)
516	የተጠቀሙባቸዉን መርፌዎች እንደገና በክዳናቸዉ ይገጥማሉ?	1. አዎ<br>2. የለም
517	የባዮሜዲካል ቆሻሻዎችን ሲያጸዱ ወይም ሲያስወግዱ ከባድ ጓንቶችን(ድዩቲ ግላብ) ይለብሳሉ?	1. አዎ<br>2. የለም
518	በጥያቄ 517 መልስዎ አዎ ከሆነ ምን ያህል ጊዜ ይለብሳሉ?	1. ሁል ጊዜ<br>2. አንዳንድ ጊዜ<br>3. በጭራሽ
519	የባዮሜዲካል ቆሻሻዎችን ሲያጸዱ ወይም ሲያስወግዱ ቦት ይለብሳሉ?	1. አዎ<br>2. የለም

520	በጥያቄ 519 መልስዎ አዎ ከሆነ ምን ያህል ጊዜ ይለብሳሉ?	1. ሁል ጊዜ<br>2. አንዳንድ ጊዜ<br>3. በጭራሽ
521	የባዮሜዲካል ቆሻሻዎችን ሲያጸዱ ወይም ሲያስወግዱ ኤፕረን(ሽርጥ) ይለብሳሉ?	1. አዎ<br>2. የለም
522	በጥያቄ 521 መልስዎ አዎ ከሆነ ምን ያህል ጊዜ ይለብሳሉ?	1. ሁል ጊዜ<br>2. አንዳንድ ጊዜ<br>3. በጭራሽ
523	እንደገና ጥቅም ላይ የሚውሉ የፅዳት መሣሪያዎችን ከእያንዳንዱ አገልግሎት በኂላ ያፀዳሉ?	1. አዎ<br>2. የለም
524	በጥያቄ 523 መልስዎ አዎ ከሆነ በምን ያጸዳሉ?<br>(ከአንድ መልስ በላይ መምረጥ ይቻላሉ)	1. በአልኮሆል<br>2. በክሎሪን<br>3. በፎርማለዳይድ<br>4. ሌላ (ይግለጹ) ___
525	ከአገልግሎት ቦታ ላይ የሚገኙ በሽታ አስተላላፊ የባዮሜዲካል ቆሻሻዎችን ሁልጊዜ ይሰበስባሉ?	1. አዎ<br>2. የለም
526	በጥያቄ 525 መልስዎ አዎ ከሆነ በምን ያህል ጊዜ?	1. በ24 ሰዓት<br>2. በ48 ሰዓት<br>3. በ72 ሰዓት<br>4. ሌላ (ይግለጹ) ---------
527	ልዩነታቸዉን መሰረት በማድረግ የባዮሜዲካል ቆሻሻዎችን በተናጠል ያጓጉዛሉ?	1. አዎ<br>2. የለም
528	በጥያቄ 527 መልስዎ አዎ ከሆነ መቸ?	1. ሁል ጊዜ<br>2. አንዳንድ ጊዜ<br>3. በጭራሽ
529	በሚያጓጉዙበት ጊዜ የባዮሜዲካል ቆሻሻ ማጠራቀሚያዎችን ይዘጋሉ?	1. አዎ<br>2. የለም
530	በጥያቄ 529 መልስዎ አዎ ከሆነ መቸ?	1. ሁል ጊዜ<br>2. አንዳንድ ጊዜ<br>3. በጭራሽ
531	የባዮሜዲካል ቆሻሻዎችን ለማጓጓዝ ምን ዓይነት መሣሪያ ይጠቀማሉ?	1. የትሮሊ / ጎማ ባሮ<br>2. የተዘጋ ባልዲ

	መቸ?(ከአንድ መልስ በላይ መልስ መምረጥ ይቻላሉ)	3. የተከፈተ ባልዲ<br>4. ሌላ (ይግለጹ) ---------
532	እጅዎን በሳሙና እና በውሃ ይታጠባሉ?	1. አዎ<br>2. የለም
533	በጥያቄ 532 መልስዎ አዎ ከሆነ መቸ? (ከአንድ መልስ በላይ መምረጥ ይቻላሉ)	1. ከታካሚ እንክብካቤ በፊት<br>2. ከታካሚ እንክብካቤ በኋላ<br>3. ከታካሚ እንክብካቤ በፊትና በኋላ<br>4. ባዮሜዲካል ቆሻሻዎችን ካጸዱ በኋላ<br>5. ሌላ (ይግለጹ) ---------
534	የደም ወይም የሰውነት ፈሳሽ የሚረጭ ሂደት በሚሠሩበት ጊዜ ዓይንን ለመከላከል መነጸር ይለብሳሉ?	1. አዎ<br>2. የለም
535	የደም ወይም የሰውነት ፈሳሽ የሚረጭ ሂደት በሚያከናወኑበት ጊዜ አፍንጫንና አፍን ለመከላከል ጭምብል ይለብሳሉ?	1. አዎ<br>2. የለም
