# Supplementary material for: Biomedical waste management practices and associated factors among health care workers in the era of the covid-19 pandemic at metropolitan city private hospitals, Amhara region, Ethiopia, 2020
Source: PLoS One. 2022 Apr 6;17(4):e0266037. doi: 10.1371/journal.pone.0266037 (PMC8985930; doi:10.1371/journal.pone.0266037)
Supplement: S2 File — (PDF) [file pone.0266037.s002.pdf]

Annex: English version Questionnaire

Please read the instruction and questions for each section before you answer, if you have unclear questions or instructions, you can ask the principal investigator or assistant. Please encircle your choice code among the given alternatives.

Section I: Socio-demographic Characteristics.		
**S.No**	**Variables**	**Answer**
101	Sex	1. Male<br>2. Female
102	Age	__________years
103	Religion	1. Orthodox<br>2. Muslim<br>3. Protestant<br>4. Catholic<br>5. Other(specify)_________
104	Marital status	1. Single<br>2. Married<br>3. Widowed<br>4. Divorced<br>5. Separated
105	Ethnicity	1. Amhara<br>2. Oromo<br>3. Afar<br>4. Tigray<br>5. 5. Others (specify)________
106	What is your level of education?	1. MSc and above<br>2. First Degree<br>3. Diploma<br>4. Certificate and bellow
107	What is your profession/job?	1. Medical doctor<br>2. nurse<br>3. Pharmacy<br>4. Medical laboratory

		5. Environmental health<br>6. Midwifery<br>7. Radiography<br>8. Anesthesia<br>9. Cleaner<br>10. Others (specify)______
108	How much is your experience as a health care worker?	______ Year/month
109	How much is your monthly salary?	_____ETB

Section II: Health care-related characteristics

S.No	Variables	Answer
201	Have you ever taken training on biomedical waste management or related issues in the facility?	1. Yes<br>2. No
202	How much is working your hours per day on your profession/job?	______
203	How much is working your day per week on your profession/job?	_______
204	In which department/ section you are working currently?	1. OPD<br>2. Ward<br>3. Laboratory<br>4. Emergency<br>5. Pharmacy<br>6. OR<br>7. Office<br>8. Others (specify)______
205	Are gloves available in sufficient quantity in the department? (sufficient means glove availability for each patient care)	1. Yes<br>2. No<br>3. Not applicable
206	Is there any guideline for biomedical waste management or infection prevention and control in the department/section?	1. Yes<br>2. 2. No

207	Is there any Standard Operating Procedure for biomedical waste management or infection prevention in the department/section?	1. Yes<br>2. No

208	Is there infectious waste stored for more than two days?	1. Yes<br>2. No
209	Does the facility use onsite BMW treatment methods?	1. Yes<br>2. 2. No
210	If yes on Q209, what kind of BMW treatment methods does it use? **(More than one answers are possible)**	1. Incineration<br>2. Sterilization<br>3. Chemical<br>4. Burning<br>5. Other (specify)
211	Are all 3 bins (black bin, yellow bin, and safety box) are available in the department/ section?	1. Yes<br>2. No
212	Are leveled bins available in the department/ section?	1. Yes<br>2. 2. No
**Section III: knowledge questions**		
**S.No**	**Questions**	**Answer**
301	Have you ever got information about biomedical waste?	1. Yes<br>2. No
302	If yes on Q401, where do you get it? (More than one answer is possible)?	1. Guideline<br>2. Training<br>3. Friends<br>4. Television<br>5. Radio<br>6. Magazine<br>7. Others (specify)
303	Have you ever got information about biomedical waste management?	1. Yes<br>2. No
304	If yes on Q403, where do you get it? (More than one answer is possible)?	1. Guideline<br>2. Training<br>3. Friends<br>4. Television<br>5. Radio<br>6. Magazine<br>7. Others (specify)

305	Have you ever got information as biomedical wastes are recyclable?	1. Yes<br>2. No
306	If yes on Q405, where do you get it? (More than one answer is possible)?	1. Guideline<br>2. Training<br>3. Friends<br>4. Television<br>5. Radio<br>6. Magazine<br>7. Others (specify)
307	Have you ever got information about the benefit of biomedical waste management?	1. Yes<br>2. No
308	If yes on Q407, where do you get it? (More than one answer is possible)?	1. Guideline<br>2. Training<br>3. Friends<br>4. Television<br>5. Radio<br>6. Magazine<br>7. Others (specify)
309	Do you know health hazards mainly associated with biomedical waste?	1. Yes<br>2. No
310	If yes on Q301, what kind of health hazard do you know? **(More than one answer is possible)**	1. HIV infection<br>2. HBV infection<br>3. HCV infection<br>4. Other (specify)
311	Does wearing personal protective equipment reduce the risk of infection?	1. Yes<br>2. No
312	If yes on Q303, what kind of risk of infection? **(More than one answer is possible)**	1. HIV<br>2. HBV<br>3. HCV<br>4. Other(specify)
313	Are all biomedical wastes biologically hazardous (infectious)?	1. Yes<br>2. No
314	Are items contaminated with body fluids considered biomedical waste?	1. Yes<br>2. No

315	Do you know as biomedical wastes are segregated into different categories at the point of generation?	1. Yes<br>2. No
316	Do you know about the color-coding of biomedical waste bins?	1. Yes<br>2. No
317	If yes on Q308, what kind of color-coding do you know? **(More than one answer is possible)**	1. Yellow bin<br>2. Black bin<br>3. Yellow safety box<br>4. Other (specify)
318	What type of biomedical waste should be stored in a yellow biomedical waste disposal bag/bin?	1. General waste<br>2. Infectious waste<br>3. I don’t know
319	What type of biomedical waste should be stored in a black biomedical waste disposal bag/bin?	1. General waste<br>2. Infectious waste<br>3. I don't know
320	What type of biomedical waste should be stored in a safety box?	1. Sharp wastes<br>2. Plastic wastes<br>3. Paper wastes
321	What is the storage of a safety box to dispose of wastes?	1. 1/2 full<br>2. 3/4 full<br>3. Full<br>4. I don’t know
322	Do you know the labeling of biomedical waste containers?	1. Yes<br>2. No
323	If yes on Q314, how do you label? **(More than one answer is possible)**	1. Infectious waste container<br>2. Non-infectious container<br>3. Sharp waste container<br>4. Other (specify)
324	Does disinfection of infectious biomedical wastes decrease infection?	1. Yes<br>2. No

325	Is there a need to close biomedical waste containers while transport?	1. Yes<br>2. No
326	Is there a need to secure stored biomedical wastes waiting for treatment or disposal?	1. Yes<br>2. No
327	What is the maximum time of infectious biomedical waste can be stored before being treated or disposed of?	1. 24 hours<br>2. 48 hours<br>3. 72 hours<br>4. I don't know
328	Do you know about BMW disposal methods?	1. Yes<br>2. No
329	If yes on Q320, what type of BMW disposal methods do you know? **(More than one answer is possible)**	1. Ash pit<br>2. Needle pit<br>3. Pit burial<br>4. Other (specify)

Section IV: Attitude questions

- Based on the following scale of measurement 1-5 ((1=Strongly Disagree (SD); 2=Disagree (D); 3= Neutral (N); 4= Agree (A) and 5=Strongly Agree (SA)); please read each statement and select your answer from the right margin of the table that you believe.

**S.No**	**What is your opinion/belief on the following statements?**	**SD**	**D**	**N**	**A**	**SA**
401	Improperly managed biomedical wastes may cause infection	1	2	3	4	5
402	Safe biomedical waste management is an issue involving the responsibilities of each health care staff	1	2	3	4	5
403	HIV may be transmitted through biomedical wastes	1	2	3	4	5
404	Hepatitis B virus may be transmitted through biomedical wastes	1	2	3	4	5
405	Hepatitis C virus may be transmitted through biomedical wastes	1	2	3	4	5

406	Biomedical wastes do not transmit any infectious diseases	1	2	3	4	5
407	Biomedical wastes should be segregated into different categories at the point of generation	1	2	3	4	5
408	Biomedical waste segregation facilitate safe handling of the waste	1	2	3	4	5
409	Labeling biomedical waste containers do not add value to biomedical waste management	1	2	3	4	5
410	Proper biomedical waste disposal is important to prevent infection transmission	1	2	3	4	5
411	Biomedical waste disinfection can reduce the chance of contracting infection	1	2	3	4	5
412	Wearing personal protective equipment helps to reduce the risk of infection	1	2	3	4	5
413	Biomedical waste management add extra burden of work	1	2	3	4	5
414	Management of biomedical waste is only the responsibility of the institution	1	2	3	4	5
415	Biohazardous wastes should be disinfected before disposal	1	2	3	4	5

Section V: Biomedical waste managmnet related question

S.No	Variables	Response
501	Have you ever encountered any sharp injury in the last 6 months?	1. Yes<br>2. No
502	If yes on Q601, what kind of sharp injury? **(More than one answer is possible)**	1. Scalpel<br>2. Blade<br>3. Needlestick<br>4. Other(specify)
503	Do you use gloves while you are handling biomedical wastes?	1. Yes<br>2. No

504	If yes on Q603, how often? (Always means use of the indicated personal protective equipment continuously while it is necessary, sometimes means when you use occasionally while it is necessary, and never means when you don't use the indicated personal protective equipment at all times while it is necessary.)	1. Always<br>2. Sometimes
505	Do you wear a gown while you are working with/handling biomedical wastes?	1. Yes<br>2. No
506	If yes on Q605, how often?	1. Always<br>2. Sometimes
507	Do you label biomedical waste containers?	1. Yes<br>2. No
508	If yes on Q607, how do you label? **(More than one answer is possible)**	1. Infectious waste container<br>2. Non-infectious container<br>3. Sharp waste container<br>4. Others(specify)__
509	Do you segregate biomedical wastes according to their type at the point of generation?	1. Yes<br>2. No
510	Do you follow color coding for the segregation of biomedical wastes?	1. Yes<br>2. No
511	If yes on Q610, what kind of color-coding do you follow? **(More than one answer is possible)**	1. Black waste bin<br>2. Yellow waste bin<br>3. Yellow safety box<br>4. Other(specify) ___
512	Where do you put non-infectious wastes like paper, plastic, and other supplies?	1. Black waste bin<br>2. Yellow waste bin<br>3. Safety box<br>4. 4.Other(specify) __
513	Where do you put infectious wastes like cotton, gauze, and other items contaminated with blood and body fluids?	1. Black waste bin<br>2. Yellow waste bin<br>3. Safety box<br>4. 4.Other (specify____

514	Where do you put sharp waste medical supplies which may cause punctures or cuts?	1. Black waste bin<br>2. Yellow waste bin<br>3. Safety box<br>4. 4. Other (specify) __
515	Where do you put expired drugs?	1. Black waste bin<br>2. Yellow waste bin<br>3. Safety box<br>4. Not applicable
516	Do you recap the used needles before disposing of?	1. Yes<br>2. No
517	Do you use heavy-duty gloves while you clean or dispose of biomedical wastes?	1. Yes<br>2. No
518	If yes on Q619, how often do you use it?	1. Always<br>2. Sometimes
519	Do you use boots while you clean or dispose of biomedical wastes?	1. Yes<br>2. No
520	If yes on Q621, how often do you use it?	1. Always<br>2. Sometimes
521	Do you use an apron while you clean or dispose of biomedical wastes?	1. Yes<br>2. No
522	If yes on Q623, how often do you use it?	1. Always<br>2. Sometimes
523	Do you disinfect/decontaminate reusable cleaning devices after each use?	1. Yes<br>2. No
524	If yes on Q625, by what means? **(More than one answers are possible)**	1. Alcohol<br>2. Chlorine bleach<br>3. Formaldehyde<br>4. Other(specify)___
525	Do you always collect infectious biomedical wastes from the service area?	1. Yes \<br>2. No

526	If yes on Q627, while do you collect?	1. Within 24 hours<br>2. Within 48 hours<br>3. Within 72 hours<br>4. Other(specify)___
527	Do you separately transport biomedical wastes according to segregation type?	1. Yes<br>2. No
528	If yes on Q629, while do you transport t?	1. Always<br>2. Sometimes
529	Do you close biomedical waste containers during transport?	1. Yes<br>2. No
530	If yes on Q631, while do you transport?	1. Always<br>2. Sometimes
531	What kind of equipment do you use to transport biomedical wastes? **(More than one answers are possible)**	1. Trolley/wheelbarrow<br>2. Closed bucket<br>3. Open bucket<br>4. Other (specify) ___
532	Do you wash your hands with soap and water during your daily activities?	1. Yes<br>2. No
533	If yes on Q631, while do you wash? **(More than one answers are possible)**	1. Before patient care<br>2. After patient care<br>3. Before and after patient care<br>4. After BMW cleaning<br>5. Other (specify) ____
534	Do you wear goggles to protect the eye during procedures that generate a spray of blood or body fluids?	1. Yes<br>2. No
535	Do you wear a mask to protect your nose and mouse during procedures that generate a spray of blood or body fluids?	1. Yes<br>2. No

Section 6: Health care facility and working department observational checklist

Data collectors should observe actual practices of health care units and tick the appropriate alternative code in the table given below.

Health Institution---------------------

Department-----------------

No	Observation	Response	
		1. Yes	2. No
701	Is there visual aid/ written instruction present near the waste receptacles?		
702	Are gloves available in sufficient quantity (sufficient means glove availability for each patient care?		
703	Are all 3 bins (black bin, yellow bin, and safety box) are available in the department/ section?<br>**If yes, continue**		
704	Does the yellow bin contain only infectious waste?		
705	Does the black bin contain only non-infectious waste?		
706	Is there a sharp waste container filled more than ¾ full?		
707	Are leveled bins available in the department/ section?		
708	Is there designated waste storage within the hospital's premises?		
709	If yes on Q708, what BMW storage method did the facility use?	1. Onsite storage room<br>2. Puncture resistant storage containers<br>3. Other(specify)_______	
710	Is there a placenta pit in the hospital?		
711	Is there a chemical disinfectant in the department?		

712	If yes, what kind of chemical?	1. Alcohol<br>2. Chlorine bleach<br>3. Formaldehyde<br>4. Other(specify)___	
713	Is there an autoclave in the hospital?		
714	Is there an autoclave in the unit?		
715	Does the facility use onsite BMW treatment methods?		
716	If yes on Q715, what type of BMW treatment method the facility use? (Multiple answers are possible)	1. Incineration<br>2. Sterilization<br>3. Chemical<br>4. Burning<br>5. Other (specify)_____	
717	Is the incinerator fenced?		
718	Does the facility use onsite BMW disposal methods?		
719	If yes on Q718 what type of infectious waste disposal method was it used? (Multiple answers are possible)	1. Ash pit<br>2. Needle pit<br>3. Pit burial<br>4. Other (specify)___	
720	Does the hospital dispose of biomedical waste outside using its vehicles?		
721	Is there any guideline for biomedical waste management?		
722	Is there any guideline for infection prevention and control in the department/section?		
723	Is there any Standard Operating Procedure for biomedical waste management or infection prevention in the department/section?		
724	Is there a designated biomedical waste management committee in the facility?		

725	Is there designated infection prevention and control Committee in the facility?		
726	Which personal protective equipment is available in this facility? (More than one answers are possible)	1. Heavy-duty gloves<br>2. Boots<br>3. Apron<br>4. Other (specify)___<br>5. None is available	
